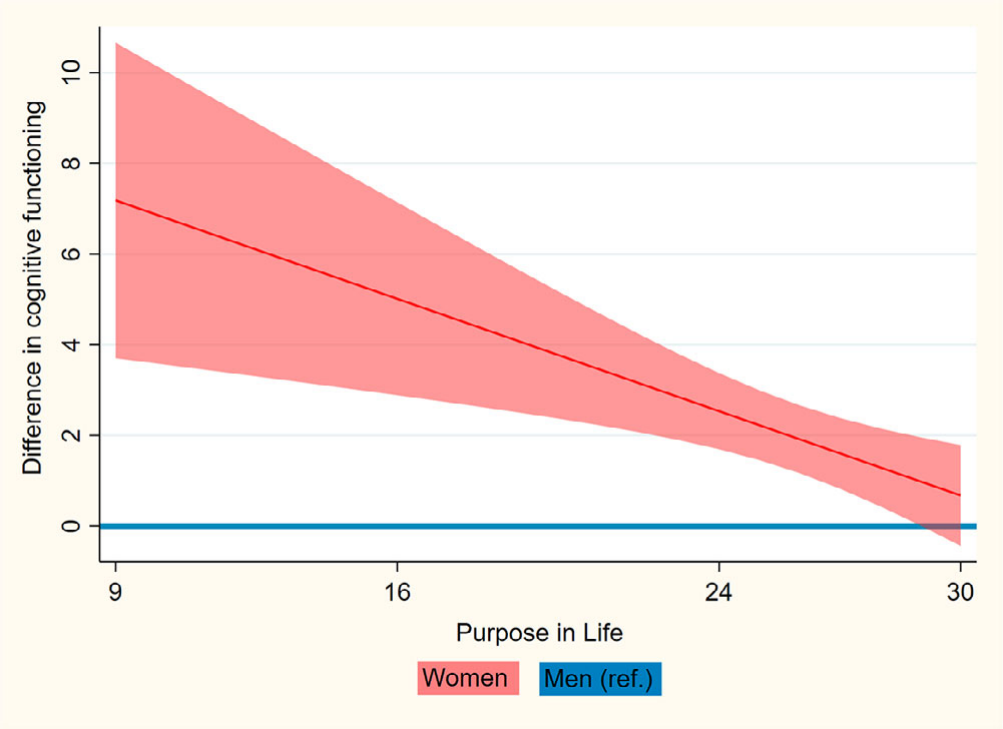# Gender difference in cognitive functioning varies by level of purpose in life – Results of the New Zealand Health, Work and Retirement (NZHWR) study

**DOI:** 10.1002/alz.091938

**Published:** 2025-01-09

**Authors:** Susanne Röhr, Rosemary Gibson, Fiona Alpass

**Affiliations:** ^1^ School of Psychology, Massey University, Auckland, Auckland New Zealand; ^2^ Global Brain Health Institute (GBHI), Trinity College Dublin, Dublin Ireland; ^3^ School of Psychology, Massey University, Palmerston North, Manawatu New Zealand

## Abstract

**Background:**

High purpose in life – the extent of engagement in activities that are personally valued and give a sense of direction and meaning to life – has been associated with higher cognitive functioning and may protect against dementia. Less is known about gender differences in cognitive functioning regarding purpose in life. Understanding gender‐specific links can inform tailored interventions aimed at promoting cognitive health.

**Method:**

A subsample (*n* = 875, aged 50‐85 years) of the NZHWR study completed face‐to‐face cognitive assessments and postal surveys in 2012. Cognitive functioning was assessed with Addenbrooke’s Cognitive Examination–Revised (ACE‐R), adapted for culturally acceptable use in New Zealand. Purpose in life was measured with the Life Engagement Test. Linear regression analysis assessed associations of gender, purpose in life and their interaction with cognitive functioning, controlling for socioeconomic factors (age, age², education, ethnicity [Māori, Indigenous people of New Zealand, and Non‐Māori, mostly of European descent], marital status, employment, individual‐level economic hardship, area‐based socioeconomic deprivation), lifestyle and health factors (smoking, alcohol consumption, physical activity, SF‐12 physical and mental health, social engagement, social loneliness).

**Result:**

The analytical sample (*n* = 643) was *M* = 65.3 (*SD* = 7.4) years old; 53.3% women, 21.2% Māori. The ACE‐R score was *M* = 92.9 (*SD* = 5.3). *N* = 55 (8.5%) scored ≥1.5*SD* below the mean, indicating cognitive impairment. Women had higher cognitive functioning (*M* = 93.7, *SD* = 4.6 vs. *M* = 92.0, *SD* = 5.8; Z = ‐3.88, *p*<.001) and purpose in life (*M* = 26.2, *SD* = 3.8 vs. *M* = 25.8, *SD* = 3.4; *Z* = ‐2.19, *p* = .029) than men. In the adjusted regression analysis (R² = 27.6%), higher purpose in life (B = 0.29, 95%CI = 0.12;0.46; *p* = .001) and female gender (B = 9.97, 95%CI = 4.71;15.24, *p*<.001) were associated with higher cognitive functioning. The association of purpose in life with cognitive functioning was less pronounced for women than men (B = ‐0.31, 95%CI = ‐0.51;‐0.11; *p* = .003) (Fig. 1). Significant covariates included age², education, deprivation, and social loneliness.

**Conclusion:**

In this sample of older New Zealanders, a gender difference in cognitive functioning varied by level of purpose in life. Women had higher cognitive functioning than men, particularly at lower purpose in life, with the difference decreasing as purpose in life increases. Interventions to enhance purpose in life might particularly benefit men. Notably, cognitive functioning may also impact purpose in life, emphasising the need for longitudinal studies.